# Precise Control of Green to Blue Emission of Halide Perovskite Nanocrystals Using Terbium Chloride as Chlorine Source

**DOI:** 10.3390/nano11092390

**Published:** 2021-09-14

**Authors:** Wenqiang Deng, Ting Fan, Jiantao Lü, Jingling Li, Tingting Deng, Mingqi Liu

**Affiliations:** 1 School of Materials Science and Hydrogen Energy, Foshan University, Foshan 528000, China; wenqiangdeng000@163.com (W.D.); lijl@fosu.edu.cn (J.L.); polop000@163.com (M.L.); 2 School of Physics and Optoelectronic Engineering, Foshan University, Foshan 528000, China; tingtingdeng0803@163.com

**Keywords:** room temperature assisted recrystallization, nanocrystalline, spectrum control

## Abstract

CsPbCl_x_Br_3-x_ nanocrystals were prepared by ligand-assisted deposition at room temperature, and their wavelength was accurately adjusted by doping TbCl_3_. The synthesized nanocrystals were monoclinic and the morphology was almost unchanged after doping. The fluorescence emission of CsPbCl_x_Br_3-x_ nanocrystals was easily controlled from green to blue by adjusting the amount of TbCl_3_, which realizes the continuous and accurate spectral regulation in the range of green to blue. This method provides a new scheme for fast anion exchange of all-inorganic perovskite nanocrystals in an open environment at room temperature.

## 1. Introduction

All inorganic cesium lead halide perovskite CsPbX_3_ (X = Cl, Br and I) quantum dots have excellent optoelectronic properties, such as excellent defect tolerance, long carrier life, a large absorption cross-section, high photoluminescence quantum yield, narrow emission peak, and adjustable emission in the visible spectrum range. It has a good application prospect in optoelectronic devices [1,2,3]. In 2015, Protesescu et al. first proposed the thermal injection method for the preparation of inorganic perovskite quantum dots [4]. By discarding the supernatant and re-dispersing in toluene or n-hexane to form a stable CsPbX_3_ nanocrystalline colloidal solution, perovskite nanocrystals with controllable morphologies, including quantum dots, nanowires, nanosheets, and nanorods, can be prepared [5,6,7,8,9]. However, this method requires the preparation of cesium oleate precursors, followed by rapid injection of lead halide precursors at high temperatures. The synthesis method requires high-temperature heating and inert gas protection, which greatly limits the application of perovskite quantum dots in practical applications.

In 2016, Li et al. prepared CsPbX_3_ nanocrystals by room-temperature ligand-assisted deposition (LARP), which is different from thermal-injection synthesis [10]. The process involves dissolving perovskite precursors, oleic acid, oleamine, etc., in a polar N, N-dimethylformamide (DMF) solvent, rather than the ODE used in the thermal injection method, and gradually adding a certain amount of the precursor solution to toluene or n-hexane under intense agitation. The salts commonly used in LARP methods are PbX_2_ and CsX. The mixing of the two solvents causes instantaneous monomer supersaturation, which triggers nucleation and growth of perovskite nanocrystals. Because the LARP process is performed in the air with a simple agitator opening, it can be scaled up to produce perovskite nanocrystals on a large scale, even up to the grade of gram [11,12]. In addition, the optical properties of CsPbX_3_ nanocrystals prepared by the room-temperature precipitation method are close to those prepared by the thermal injection method, although the reaction temperature is low and there is no inert atmosphere to protect the nanocrystals [13,14,15,16].

Different from traditional semiconductor quantum dots, the average size of CsPbX_3_ nanocrystals (NCs) is usually larger than 10 nm, larger than its exciton Bohr radius, so it is difficult to achieve continuous fluorescence emission only through size adjustment [17,18,19]. Thanks to the unique anion exchange characteristics and high ion mobility of CsPbX_3_ nanocrystals, fluorescence emission wavelength tuning of full visible-spectrum emission can be achieved by exchanging nanocrystalline halogen ions (Cl, Br or I) [20,21,22,23]. However, the traditional anion exchange reaction using PbCl_2_ needs to undergo complex pretreatment, excessive ligand-induced degradation of nanocrystals, and incomplete ionization of PbCl_2_ salts. These will result in unpredictable fluorescence shifts and cannot be tuned quantitatively and accurately [24]. For example, if excessive oleamine (OLA) is used, CsPbBr_3_ NCs will be partially or completely converted to Cs_4_PbBr_6_ NCs [25]. The addition of alkyl ammonium bromide in the anion exchange process will also lead to the unexpected evolution of the CsPb_2_Br_5_ perovskite phase. In this regard, the key to accurate emission control of CsPbX_3_ NCS is to establish a programmed and accurate anion exchange route with high halide ion reactivity to prepare pure phase CsPbX_3_ NCs [26]. In the anion exchange process, some efforts have been made to improve the reactivity of halides. Benzoyl halides and trimethylsilyl halides are electrophilic reagents. They show high reactivity in the anion exchange reaction by destroying halogen bonds, releasing halide ions, and promoting the substitution of halide components in CsPbX_3_ NCS, although these reagents are toxic [27,28]. The environmentally friendly metal halide solid MX_2_ (M = Zn, Ni and Mg) is also used as a halide source to achieve rapid anion exchange at room temperature [29,30]. The inorganic halide salt greatly simplifies the reaction process and speeds up the reaction rate. However, in the process of inorganic halide exchange, with the opening of rigid halide octahedron structure around lead, partial cation exchange of metal cations to lead is likely to occur. Different from the traditional anion exchange method, in 2019, Liu et al. dissolved ZnX_2_ in water, and the prepared ZnX_2_ aqueous solution was used as the halide source in anion exchange. The fluorescence emission peak position of CsPbX_3_ nanocrystals can be adjusted continuously and accurately in the whole visible spectrum [25]. However, due to the introduction of water, the nanocrystals will inevitably dissociate. In 2020, Pan et al. introduced a NiCl_2_ solution into the CsPbBr_3_ reaction medium, where the emission wavelength was in the range of 508–432 nm and its PLQY reached 89%. However, Ni^2+^ inevitably entered the Pb lattice, resulting in changes in the structure, thus affecting its optical properties and uncontrollable factors for accurately regulating the fluorescence spectrum band [30].

All-inorganic mixed halide perovskite is often used to obtain blue LEDs with shorter emission wavelengths. For example, Zheng and his collaborators recently reported the preparation of all-inorganic CsPb(Cl/Br)_3_ Perovskite blue led by the thermal injection method, and obtained perovskite lead with a luminous wavelength of 470 nm and efficiency of 6.3% [31]. In this study, we reported the successful synthesis of CsPbCl_x_Br_3-x_ nanocrystals at room temperature by an improved supersaturated reprecipitation method by introducing different amounts of TbCl_3_ solution into the reaction medium. The fluorescence emission peak position of the synthesized CsPbCl_x_Br_3-x_ nanocrystals is adjustable in the range of 431 nm to 512 nm and has good phase/chemical stability. Experiments show that the fluorescence emission of CsPbX_3_ nanocrystals can be accurately and continuously regulated like traditional semiconductor nanocrystals, making perovskite nanocrystals more competitive in lighting and display applications.

## 2. Experiments

### 2.1. Experimental Materials

The experimental materials include cesium bromide (CsBr, analytically pure, purity 99.5%); PbBr_2_ (analytically pure, purity 99.0%), terbium trichloride, hexahydrate (TbCl_3_.6H_2_O, analytically pure, purity 99.9%); Oleic acid (purity 85%); Oleylamine (80~90% purity); N,N-dimethylformamide (DMF, analytically pure, 99.5%); and toluene, all of which were not further purified.

### 2.2. Synthesis of CsPbCl_x_Br_3-x_ Nanocrystalline

The all-inorganic perovskite CsPbBr_3_ was synthesized by room-temperature supersaturation recrystallization. Under the condition of magnetic stirring and heating, 0.4 mmol CsBr, 0.4 mmol PbBr_2_, 1 mL oleic acid, and 0.5 mL oleylamine were dissolved in 10 mL DMF as the sources of Cs, Pb, and Br. Only 1 mL of the PbBr_2_/CsBr solution was slowly injected into 10 mL of toluene under ultrasonic conditions. After a few seconds, bright green light emission was observed under UV irradiation of 365 nm.

Synthesis of CsPbCl_x_Br_3-x_ nanocrystals: Firstly, 1 mmol TbCl_3_.6H_2_O was dissolved in 1 mL DMF as a Cl source under magnetic stirring. Then 5, 10, 20, 40, and 80 μL TbCl_3_ solutions were added dropwise, followed by 1 mL of the above-mentioned PbBr_2_/CsBr mixture solution and 10 mL of toluene. The process was carried out under ultrasound. After a few seconds, green and blue light emission was observed under the irradiation of an ultraviolet ray at 365 nm. The nanocrystals with the addition of 5, 10, 20, 40, and 80 μL TbCl_3_ solutions represent CsPbCl_0.33_Br_2.67_, CsPbCl_0.6_Br_2.4_, CsPbCl_1_Br_2_, CsPbCl_1.5_Br_1.5_, and CsPbCl_2_Br_1_, respectively.

### 2.3. Purifications

The nanocrystals synthesized at room temperature were centrifuged at 3000 r/min for 5 min, and the supernatant was retained. Then, the supernatant was centrifuged at 11,000 r/min for 15 min, and 3 mL toluene was added to the resulting precipitate, which was further centrifuged at 11,000 r/min for 15 min. The precipitate was retained, and finally it was dispersed into 3 mL toluene for optical performance testing and morphology characterization.

### 2.4. Characterizations

X-ray powder diffraction image (XRD) shows CuKα emission (λ = 1.5418 A) assisted by the Bruker Smart-Apex-II X-Ray Single-Crystal Diffractometer. Transmission Electron Microscope Hitachi H-800 (acceleration voltage of 200 kV) is used for transmission electron microscope image photography. UV-Vis absorption spectra were characterized by the Shimazu -3600 UV-NIR spectrophotometer. The Horiba Fluoromax-4 steady-state and transient fluorescence spectrometer measured the emission spectrum and fluorescence lifetime. X-ray photoelectron spectroscopy (XPS) was measured by the Thermo ESCALAB 250XI spectrometer with Al Ka excitation (1486.6 eV).

## 3. Results and Discussions

**Figure 1:**
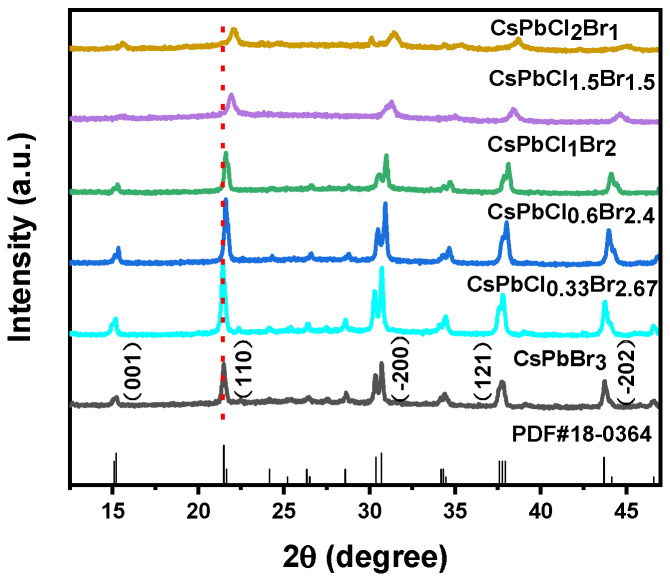
XRD patterns of CsPbClxBr3-x nanocrystals with different halogen components.

As can be seen from Figure 1, the diffraction peaks 15.081, 21.498, 30.698, 37.603, and 43.692 of CsPbCl_x_Br_3-x_ nanocrystals with different halogen components correspond to (001), (110), (−200), (121), and (−202) crystal planes of CsPbBr_3_ (PDF#18-0364) standard card, respectively. Therefore, the nanocrystals possess the crystalline structure of monoclinic bulk CsPbBr_3_ (PDF#18-0364) [30]. With the increase of Cl^−^ ion content, the above diffraction peaks gradually move to a large angle, which is caused by the lattice shrinkage caused by the replacement of Br^−^ (1.96 Å) ions with a larger radius to smaller Cl^−^ (1.81 Å) ions [32,33]. For the CsPbX_3_ NCs, monoclinic is a metastable structure, the formation of which is thermodynamically controlled. In the room-temperature ligand-assisted deposition, the reaction temperature is rather low that the total energy is insufficient to overcome the barrier to crystallizing into the tetragonal phase as can be obtained in the hot-injection approach. The existence of the stable metastable phase at low temperature is attributed to the capping ligands (OLA) on the surface of the as- prepared NCs, which lower the surface energy [34].

**Figure 2:**
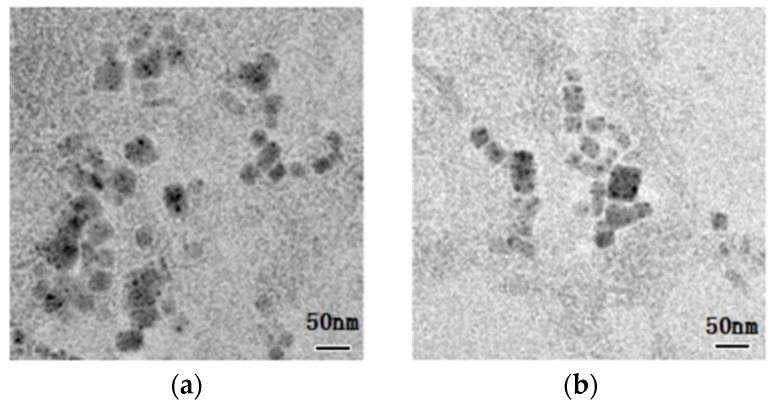
(**a**) TEM images of CsPbBr_3_ nanocrystals and (**b**) CsPbCl_1_Br_2_ nanocrystals.

Figure 2a,b shows TEM images of CsPbBr_3_ and CsPbCl_1_Br_2_ nanocrystals prepared by supersaturated recrystallization at room temperature. It can be seen from the figures that CsPbBr_3_ and CsPbCl_1_Br_2_ nanocrystals maintain uniform distribution and exhibit good monodispersion properties. The size of most of them is about 20 nm, showing high dimensional uniformity, and their morphology almost does not change before and after doping.

**Figure 3:**
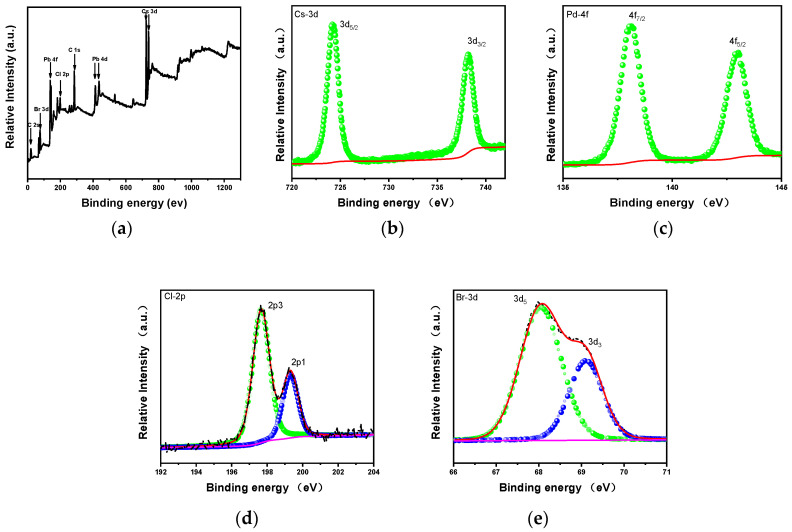
(**a**) The XPS spectrum of CsPbCl_1_Br_2_ nanocrystals; (**b**) XPS spectra of Cs 3d in CsPbCl_1_Br_2_ nanocrystals; (**c**) XPS spectra of Pb 4f in CsPbCl_1_Br_2_ nanocrystals; (**d**) XPS spectra of Cl 2p in CsPbCl_1_Br_2_ nanocrystals; (**e**) XPS spectra of Br 3d in CsPbCl_1_Br_2_ nanocrystals.

From the XPS spectrum of Figure 3a–e, the characteristic peaks of Cs 3d, Pb 4d, Pb 4f, Br 3d, and Cl 2p can be clearly observed, among which the characteristic peak of Cl 2p appears, further indicating that the characteristic peak of Tb^3+^ still does not appear in the crystal lattice position of bromine successfully incorporated by the chloride ion under the detection limit of XPS measurement. This means that Tb^3+^ will not be mixed into the lattice position of CsPbCl_x_Br_3-x_. This not only avoids the pollution of metal ions when metal halides are used as the halogen source, but also solves the problems of incomplete ionization and toxicity of Pb ions when PbCl_2_ is traditionally used as the halogen source [35].

**Figure 4:**
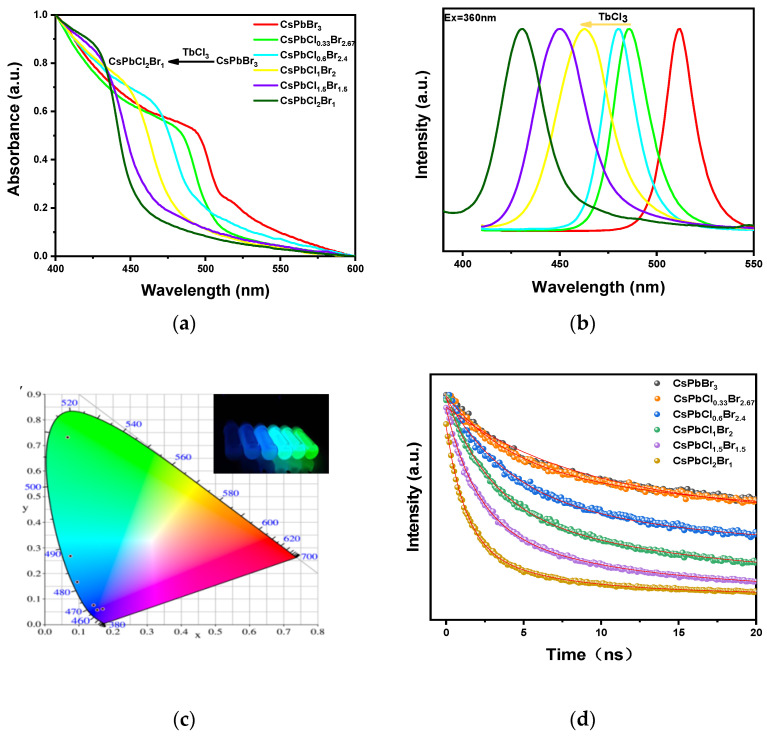
(**a**) Ultraviolet absorption spectra of CsPbCl_x_Br_3-x_ nanocrystals with different halogen components; (**b**) the fluorescence emission spectra of CsPbCl_x_Br_3-x_ nanocrystals with different halogen components (from left to right are CsPbCl_2_Br_1_, CsPbCl_1.5_Br_1.5_, CsPbCl_1_Br_2_, CsPbCl_0.6_Br_2.4_, CsPbCl_0.33_Br_2.67_, and CsPbBr_3_). (**c**) The CIE diagram of different halogen components of CsPbCl_x_Br_3-x_ nanocrystals. The illustration shows the photos of the synthesized CsPbCl_x_Br_3-x_ nanocrystals under the ultraviolet lamp; (**d**) fluorescence lifetime diagrams of CsPbCl_x_Br_3-x_ nanocrystals with different halogen components.

In order to observe the influence of chloride ion doping on the band gap of CsPbCl_x_Br_3-x_ nanocrystals, we measured the absorption spectra of CsPbCl_x_Br_3-x_ nanocrystals after adding different amounts of TbCl_3_, as shown in Figure 4a. It can be seen from the figure that, with the increase of TbCl_3_ addition, the absorption edge of CsPbCl_x_Br_3-x_ moves towards the short-wavelength direction, which is mainly caused by the increase of the band gap of CsPbCl_x_Br_3-x_ nanocrystals as Cl^−^ replaces Br^−^, corresponding to the blue shift of the fluorescence emission peak position [36]. As shown in Figure 4b, fluorescence emission can be adjusted from 512 nm to 431 nm only by changing the content of doped TbCl_3_.The nanocrystals can be controlled continuously and accurately from green light to blue light, and the half-peak width of the emission spectrum is very narrow, ranging from 18.1 nm to 34.1 nm. As shown in the inset of Figure 4c, with the increase of TbCl_3_ addition, the luminescence of the corresponding CsPbCl_x_Br_3-x_ nanocrystals under ultraviolet light gradually changes from green to blue. After two weeks, the luminescence brightness of the colloidal dispersion of CsPbX_3_ nanocrystals remained basically unchanged. The corresponding CIE color coordinates are (0.066, 0.732), (0.066, 0.732), (0.094, 0.166), (0.142, 0.076), (0.154, 0.057), and (0.169, 0.061), respectively. Interestingly, we found similar results when using EuCl_3_ as the chlorine source, which could be seen in Figures S1 and S2.

As can be seen in Figure 4d, the change of halogen components will lead to the change of average fluorescence lifetime. The overall performance is as follows: With the increase of Cl^−^ ion content, the fluorescence lifetime will be shortened, and the band gap corresponding to halogen ions is negatively correlated with the attenuation lifetime [37]. When fitting the fluorescence decay lifetime spectrum of CsPbCl_x_Br_3-x_, it is found that the fluorescence decay process of the sample conforms to the double exponential decay, and its average fluorescence lifetime is calculated by the following formula:

$$\tau_{\mathrm{av}}=\frac{\left(\alpha_{1}\tau_{1}^{2}+\alpha_{2}\tau_{2}^{2}\right)}{\left(\alpha_{1}\tau_{1}+\alpha_{2}\tau_{2}\right)}$$

Among them, τ_i_ and α_i_ are the weight coefficient and fluorescence lifetime coefficient of PL curve life, respectively. The fluorescence lifetime of CsPbBr_3_ nanocrystals is up to 65.5 ns. By fitting the fluorescence attenuation lifetime spectrum of CsPbCl_x_Br_3-x_, it is found that its fluorescence attenuation process conforms to the three-exponential decay, and its average fluorescence lifetime is calculated by the following formula:

$$\tau_{\mathrm{av}}=\frac{\alpha_{1}\tau_{1}^{2}+\alpha_{2}\tau_{2}^{2}+\alpha_{3}\tau_{3}^{2}}{\alpha_{1}\tau_{1}+\alpha_{2}\tau_{2}+\alpha_{3}\tau_{3}}$$

The fluorescence lifetime of CsPbCl_0.33_Br_2.67_, CPbCl_0.6_Br_2.4_, CsPbCl_1_Br_2_, CsPbCl_1.5_Br_1.5_, and CsPbCl_2_Br_1_ were 56.9 ns, 38.7 ns, 17.9 ns, 8.7 ns, and 4.3 ns, respectively. The luminescence data of CsPbCl_x_Br_3-x_ nanocrystals can be seen in Table 1.

## 4. Conclusions

In this paper, CsPbCl_x_Br_3-x_ nanocrystals were directly synthesized by doping with TbCl_3_ at room temperature in an open environment. The synthesized CsPbCl_x_Br_3-x_ nanocrystals belong to the monoclinic phase and maintain uniform distribution, showing good monodispersion properties. The size of most of them is about 20 nm and their morphology almost does not change before and after doping. By adjusting the amount of TbCl_3_, the fluorescence emission was accurately adjusted from green to blue. This provides a new scheme for fast anion exchange of perovskite nanocrystals with all-inorganic cesium-lead halide in an open environment at room temperature and has practical application value in the fields of light-emitting diodes, solar cells, and photodetectors.

## Figures and Tables

**Table 1:** Luminescence data of CsPbCl_x_Br_3-x_ nanocrystals based on different halogen components.

The Sample	The Emission Peak	Full Width at Half Height	Mean Fluorescence Lifetime	CIE Coordinates
CsPbBr_3_	512 nm	18.1 nm	65.5 ns	(0.066, 0.732)
CsPbCl_0.33_Br_2.67_	486 nm	23.1 nm	56.9 ns	(0.075, 0.269)
CsPbCl_0.6_Br_2.4_	480 nm	20.7 nm	38.7 ns	(0.094, 0.166)
CsPbCl_1_Br_2_	463 nm	34.1 nm	17.9 ns	(0.142, 0.076)
CsPbCl_1.5_Br_1.5_	450 nm	32.3 nm	8.7 ns	(0.154, 0.057)
CsPbCl_2_Br_1_	431 nm	27.3 nm	4.3 ns	(0.169, 0.061)

## Data Availability

The data presented in this study are available in this paper.

## Supplementary Materials

The following are available online at https://www.mdpi.com/article/10.3390/nano11092390/s1, Figure S1: Photo of CsPbCl_x_Br_3-x_ nanocrystals under ultraviolet light (from left to right, CsPbBr_3_, CsPbCl_0.15_Br_2.85_, CsPbCl_0.27_Br_2.73_, CsPbCl_0.4_Br_2.6_, CsPbCl_0.82_Br_2.18_, CsPbCl_1.16_Br_1.84_ and CsPbCl_1.5_Br_1.5_, respectively). Figure S2: Fluorescence emission spectra of CsPbCl_x_Br_3-x_ nanocrystals with different halogen components (from left to right, CsPbCl_1.5_Br_1.5_, CsPbCl_1.16_Br_1.84_, CsPbCl_0.82_Br_2.18_, CsPbCl_0.4_Br_2.6_, CsPbCl_0.27_Br_2.73_, CsPbCl_0.15_Br_2.85_ and CsPbBr_3_, respectively).
