# Supplementary material for: Precise Control of Green to Blue Emission of Halide Perovskite Nanocrystals Using Terbium Chloride as Chlorine Source
Source: Nanomaterials (Basel). 2021 Sep 14;11(9):2390. doi: 10.3390/nano11092390 (PMC8470515; doi:10.3390/nano11092390)
Supplement: Supplementary file 1 [file nanomaterials-11-02390-s001.zip › nanomaterials-1329895-supplementary.pdf]

nanomaterials

*Supplementary Material*

# Precise Control of Green to Blue Emission of Halide Perovskite Nanocrystals Using Terbium Chloride as Chlorine Source

**Wenqiang Deng [1], Ting Fan [1,*], Jiantao Lü [2,*], Jingling Li [1], Tingting Deng [2] and Mingqi Liu [1]**

[1] School of Materials Science and Hydrogen Energy, Foshan University, Foshan 528000, China; wenqiangdeng000@163.com (W.D.); lijl@fosu.edu.cn (J.L.); polop000@163.com (M.L.)
[2] School of Physics and Optoelectronic Engineering, Foshan University, Foshan 528000, China; tingtingdeng0803@163.com
* Correspondence: fanting@fosu.edu.cn (T.F.); keentle@gmail.com (J.L.)

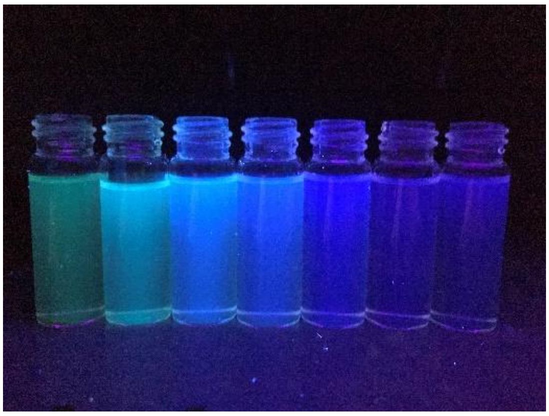

**Figure S1.** Photo of $CsPbCl_xBr_{3-x}$ nanocrystals under ultraviolet light (from left to right, $CsPbBr_3$, $CsPbCl_{0.15}Br_{2.85}$, $CsPbCl_{0.27}Br_{2.73}$, $CsPbCl_{0.4}Br_{2.6}$, $CsPbCl_{0.82}Br_{2.18}$, $CsPbCl_{1.16}Br_{1.84}$ and $CsPbCl_{1.5}Br_{1.5}$, respectively.).

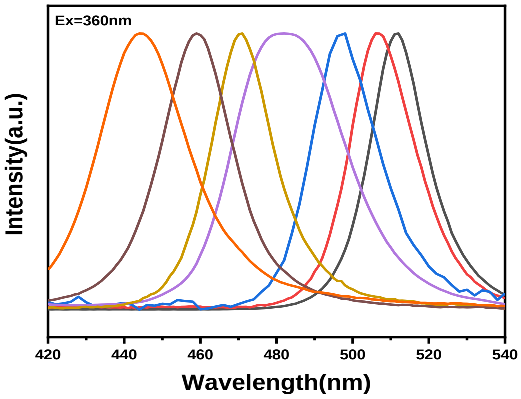


**Figure S2.** Fluorescence emission spectra of $CsPbCl_xBr_{3-x}$ nanocrystals with different halogen components (from left to right, $CsPbCl_{1.5}Br_{1.5}$, $CsPbCl_{1.16}Br_{1.84}$, $CsPbCl_{0.82}Br_{2.18}$, $CsPbCl_{0.4}Br_{2.6}$, $CsPbCl_{0.27}Br_{2.73}$, $CsPbCl_{0.15}Br_{2.85}$ and $CsPbBr_3$, respectively.).
